# Calpain-5 Mutations Cause Autoimmune Uveitis, Retinal Neovascularization, and Photoreceptor Degeneration

**DOI:** 10.1371/journal.pgen.1003001

**Published:** 2012-10-04

**Authors:** Vinit B. Mahajan, Jessica M. Skeie, Alexander G. Bassuk, John H. Fingert, Terry A. Braun, Heather T. Daggett, James C. Folk, Val C. Sheffield, Edwin M. Stone

**Affiliations:** 1 Department of Ophthalmology and Visual Sciences, University of Iowa, Iowa City, Iowa, United States of America; 2 Department of Pediatrics, University of Iowa, Iowa City, Iowa, United States of America; 3 Department of Neurology, University of Iowa, Iowa City, Iowa, United States of America; 4 Department of Biomedical Engineering, University of Iowa, Iowa City, Iowa, United States of America; 5 Howard Hughes Medical Institute, Iowa City, Iowa, United States of America; Stanford University School of Medicine, United States of America

## Abstract

Autosomal dominant neovascular inflammatory vitreoretinopathy (ADNIV) is an autoimmune condition of the eye that sequentially mimics uveitis, retinitis pigmentosa, and proliferative diabetic retinopathy as it progresses to complete blindness. We identified two different missense mutations in the *CAPN5* gene in three ADNIV kindreds. *CAPN5* encodes calpain-5, a calcium-activated cysteine protease that is expressed in retinal photoreceptor cells. Both mutations cause mislocalization from the cell membrane to the cytosol, and structural modeling reveals that both mutations lie within a calcium-sensitive domain near the active site. *CAPN5* is only the second member of the large calpain gene family to cause a human Mendelian disorder, and this is the first report of a specific molecular cause for autoimmune eye disease. Further investigation of these mutations is likely to provide insight into the pathophysiologic mechanisms of common diseases ranging from autoimmune disorders to diabetic retinopathy.

## Author Summary

We care for several families with an inherited form of autoimmune inflammation inside the eye. The patients also develop bleeding, scar tissue, and eventually blindness. Using advanced gene analysis methods, we discovered the cause of this disease is gene mutations in the *CAPN5* gene. This gene makes a protein, calpain-5, which belongs to a family of calcium-activated enzymes that slice other proteins inside cells. Calpain-5 is expressed in the retina, and the disease mutations alter its location inside the cell. Future studies to understand how this protein causes inflammation and bleeding inside the eye will help us develop treatments for this condition and more common eye diseases with inflammation and bleeding.

## Introduction

Autosomal dominant neovascular inflammatory vitreoretinopathy (ADNIV) is a heritable autoimmune condition. It is characterized by various stages that mimic several much more common eye diseases, including: uveitis, retinitis pigmentosa, proliferative diabetic retinopathy and proliferative vitreoretinopathy [1], [2]. Together, these diseases account for a significant fraction of visual morbidity and human blindness [3], [4]. Identification of a gene that generates the varied pathological features of these common conditions could have a significant impact on the understanding and treatment of blindness [5]. Although there are numerous causative genes for retinitis pigmentosa, only a handful of genes have previously been associated with intraocular inflammation, neovascularization and fibrotic disease [6].

**Figure 1:**
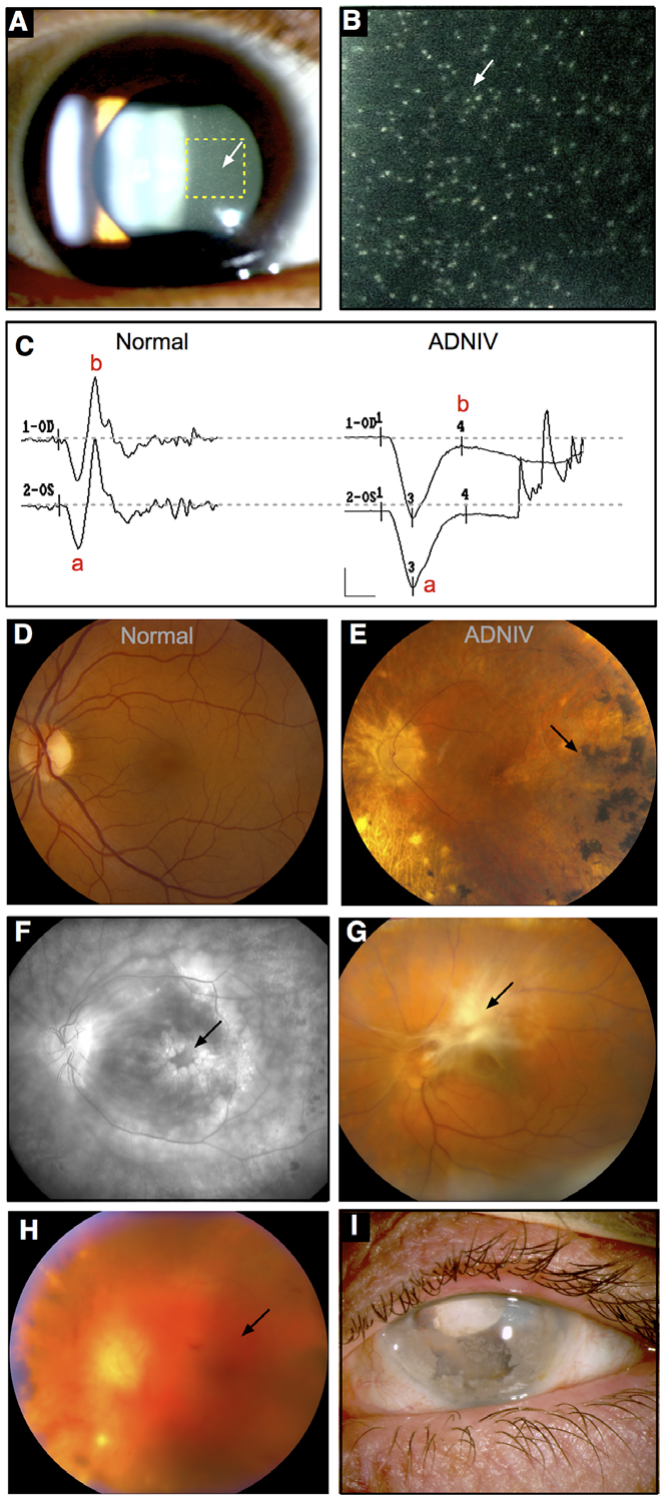
ADNIV phenotype. A–B. Clusters of autoimmune reactive leukocytes are visible in the vitreous cavity (inset, arrows). C. Electroretinography shows loss of the b-wave. D. Fundus image of the normal retina. E. Fundus image of the ADNIV retina shows pigmentary degeneration (arrow) similar to retinitis pigmentosa. F. Fluorescein angiography reveals cystoid macular edema at the fovea (arrow), a consequence of autoimmune intraocular inflammation. G. Preretinal fibrosis leads to tractional retinal detachments. H. Vitreous hemorrhage (arrow) from retinal neovascularization. I. Phthisis bulbi and involution of eye tissues in end-stage ADNIV.

Because of its similarity to other common eye diseases, ADNIV patients are often misdiagnosed, unless the familial nature of their disease is recognized. Bennett and co-workers described the original ADNIV family, ADNIV-1 in this study [1], in which the characteristic clinical findings were transmitted in an autosomal dominant fashion through eight-generations. The disease onset in this family varies between 10 and 30 years of age and the disease course can be divided into five stages, each lasting approximately ten years (Figure 1, Figure S1) [2]. In the first stage, ADNIV is clinically indistinguishable from an autoimmune, non-infectious uveitis [7]. Although the retina appears normal, an abnormality is detectable with electroretinography very early in the course of the disease. In the second stage, retinitis pigmentosa-like photoreceptor degeneration is apparent. In the third stage, retinal neovascularization develops, which is very similar to the pathologic angiogenesis of proliferative diabetic retinopathy [8]. In the fourth stage, intraocular fibrosis leads to retinal detachment, similar to that seen in proliferative vitreoretinopathy [9]. In the fifth stage, continued inflammation, intraocular hemorrhage, neovascular glaucoma, fibrosis and retinal detachment eventually lead to phthisis and complete blindness. There are no systemic features in this condition. This combination of overlapping clinical conditions is unusual and suggests that the disease-causing mutations may act through multiple pathways.

Stone and co-workers previously mapped the genetic locus for ADNIV to chromosome 11q13 [10]. In this study, we identified two new ADNIV families, and these additional subjects provided an opportunity to refine the genetic interval and identify the causative gene. Two different mutations were identified among three ADNIV families in the gene encoding calpain-5, an intracellular calcium-activated cysteine protease with an unknown physiological function.

## Results

### Clinical Findings

**Figure 2:**
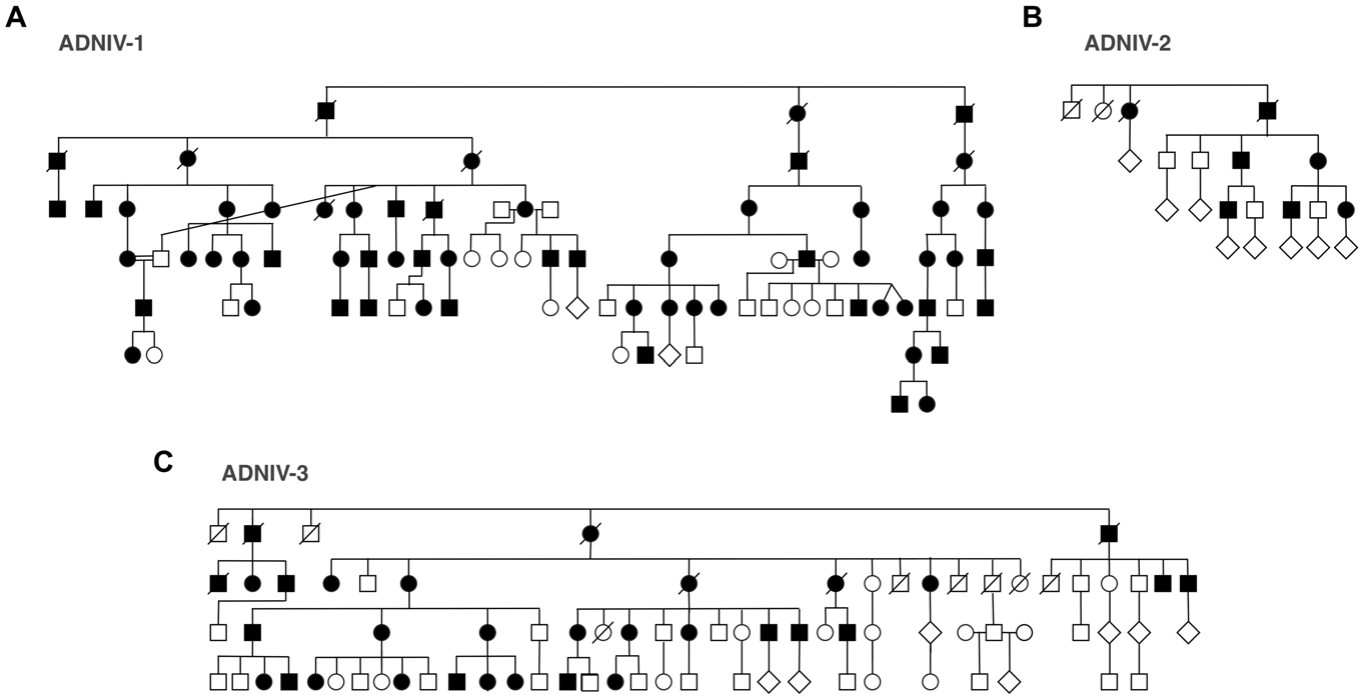
ADNIV pedigrees. A–C. Three families with clinical features of ADNIV exhibit a dominant pattern of inheritance. Black symbols represent clinically affected subjects. Open symbols represent unaffected subjects. Deceased individuals are marked by a slash.

The new ADNIV families displayed a phenotype very similar to the original ADNIV-1 pedigree. Specifically, affected members showed all of the previously reported clinical signs of the disease (Figure 1, Figure S1) including: non-infectious uveitis (Figure 1A, 1B), early loss of the b-wave on electroretinography (Figure 1C), pigmentary retinal degeneration (Figure 1D, 1E), cystoid macular edema, (Figure 1F), retinal and iris neovascularization, vitreous hemorrhage (Figure 1H), epiretinal membrane formation, proliferative vitreoretinopathy (Figure 1G), retinal detachment, cataract, neovascular glaucoma and ultimately phthisis and complete blindness (Figure 1I) [1]. Each pedigree was consistent with autosomal dominant inheritance with complete penetrance (Figure 2). There were sixty-one affected subjects in ADNIV-1, seven in ADNIV-2, and thirty-one in ADNIV-3 (Figure 2). Forty-two of these 99 affected individuals (42%) were male. The clinical severity of the disease was indistinguishable between affected males and females. With the exception of psoriasis in one individual, there were no other systemic, autoimmune or inflammatory conditions present in any of the affected family members.

### Linkage Analysis

**Figure 3:**
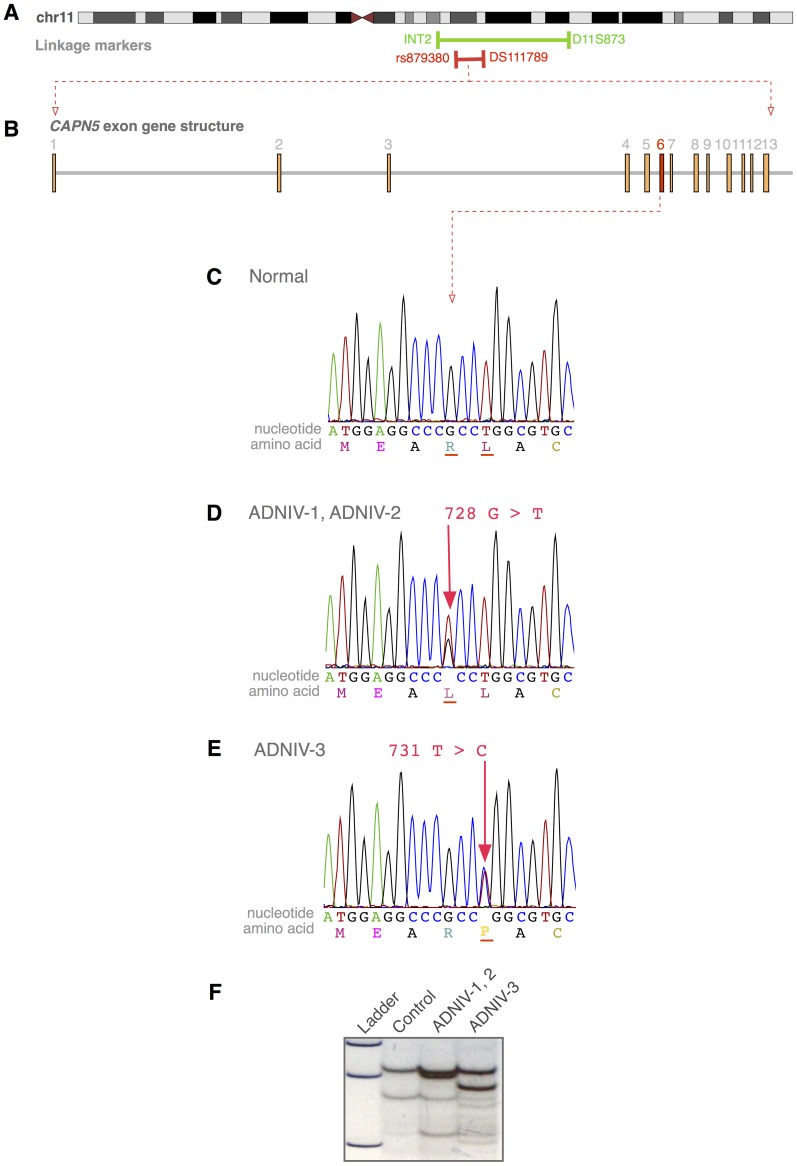
The *CAPN5* gene harbors mutations in exon 6 of ADNIV subjects. A. The ADNIV locus was previously mapped to chromosome 11q13 (green bar). STRP and SNP array mapping narrowed the interval (red bar). B. *CAPN5* gene structure is composed of 13 exons. C. Chromatogram of normal *CAPN5* DNA sequence in exon 6. D–E. Chromatograms of ADNIV subjects shows mutations in *CAPN5* exon 6. F. SSCP distinguishes between normal sequence and ADNIV mutations.

Prior linkage analysis of the ADNIV-1 family mapped the disease-causing mutation to a 22-megabase (chr11: 91,760,018–69,339,635) interval on chromosome 11q13 (Figure 3A) [10]. Genotyping of the ADNIV-2 and ADNIV-3 families with short tandem repeat polymorphisms was consistent with linkage to the same locus. Haplotype analysis was suggestive of an ancestral relationship between ADNIV-1 and ADNIV-2. In addition, two affected individuals in the ADNIV-3 family were found to be recombinant within the disease interval, narrowing it to 6.5 megabases between D11S4139 and D11S1789. High resolution SNP genotyping of ADNIV-1 and ADNIV-3 further reduced the interval to the 6 megabases between rs879380 and D11S1789, a region harboring 86 known genes (Figure 3A).

### Exome Sequencing

Whole-exome sequencing was performed using DNA from two affected family members from the ADNIV-1 pedigree who were separated by seven meioses. Only one of the resultant sequence variations met the following four criteria: located within the 6 Mb ADNIV interval, shared by the two affected members of ADNIV-1, not previously reported as a polymorphism, and nonsynonymous. This variant is a guanine to thymine nucleotide change (c.728G>T, p.Arg243Leu) in exon 6 of the *CAPN5* gene (NM_004055) (Figure 3B–3D). A combination of SSCP and Sanger sequencing of *CAPN5* exon 6 verified that this mutation was present in all the affected members and none of the unaffected members of ADNIV-1.

The coding sequence of *CAPN5* was then sequenced in affected members of the two other ADNIV pedigrees. All affected members of ADNIV-2 were found to harbor the same heterozygous variant (c.728G>T, p.Arg243Leu) found in ADNIV-1, supporting the suspected ancestral relationship between these two families (Figure 3D). All affected members of the ADNIV-3 family, were found to harbor a heterozygous variant in the adjacent codon, a thymine to cytosine change (c.731T>C, p.Leu244Pro) (Figure 3E). Both of these putative disease-causing variants in exon 6 of *CAPN5* were easily detectable by SSCP in ADNIV patients, but were absent from all unaffected adult members of the ADNIV families (no asymptomatic minors were tested) as well as 272 ethnically similar control individuals (Figure 3F). None of the three variants were listed in the dbSNP or 1000 Genome databases. In addition, none of the variant alleles were found in the over 10,700 *CAPN5* alleles sequenced in the NHLBI Exome Sequencing Project (http://evs.gs.washington.edu/EVS/)

### Structural Analysis of Calpain-5 ADNIV Mutations

**Figure 4:**
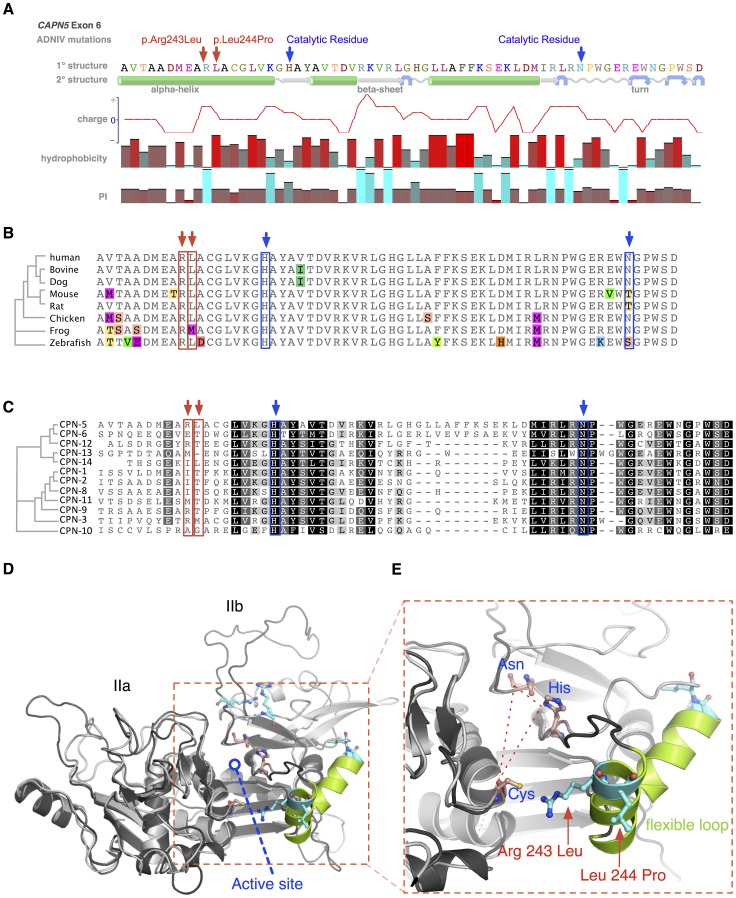
Protein structure modeling of calpain-5 and ADNIV mutants. A. Both ADNIV mutations (red arrows) are located in exon 6, which encodes a portion of the catalytic domain and two of three catalytic residues (blue arrows). Primary protein sequence analysis shows the ADNIV-1/2 and ADNIV-3 mutations to be 8–9 amino acids upstream of the catalytic histidine residue. Secondary structure modeling shows that the two mutations are within a putative alpha helical domain. One mutated codon results in the loss of a basic residue, while the other introduces a proline into the putative alpha helix. B. Alignment of calpain-5 orthologs shows very high evolutionary conservation of the mutated residues (red arrows). Amino acid mismatches are color-highlighted. C. Twelve human calpain paralogs show significant differences in exon 6 (Black, 100% similarity; Dark grey, 80–100% similarity; Light grey, 60–80% similarity; White, less than 60% similarity). D. Three-dimensional modeling of the catalytic domain shows the location of the active site cleft (red outline). E. Magnified view of the active site cleft shows the catalytic triad (dashed line – blue text) and location of the two mutations (red arrows and text). Both mutations are located within a peptide loop that is homologous to a flexible loop of calpain-1 that undergoes a calcium-induced conformational change in association with regulation of the active site cleft (see text).

Calpain-5 is an intracellular calcium-activated cysteine protease (NP_004046) with evolutionarily conserved domains required for protease activity. Both ADNIV-causing mutations were found in exon 6, which encodes a major part of the catalytic domain and contains two of the three catalytic residues that compose the active site (Figure 4). Modeling of secondary structure suggests that both the ADNIV-1/2 (p.Arg243Leu) and ADNIV-3 (p.Leu244Pro) mutations lie within a nearby alpha helical domain. The first of these mutations removes a charged residue while the second disrupts the putative helical structure (Figure 4A).

The amino acid sequence of *CAPN5* exon 6 is highly conserved across vertebrate species (Figure 4B). The catalytic residues show 100% conservation among *CAPN5* orthologs. Interestingly, there is also 100% conservation of the Arg243 residue mutated in ADNIV-1/2 and 88% conservation of the Leu244 mutated in ADNIV-3. This small evolutionary divergence at the latter codon is also quite conservative: methionine for leucine in the frog. In contrast, the disease-causing mutation at this codon introduces a new proline bend within a putative alpha helix.

Previous comparisons of calpain-5 to its human paralogs demonstrated that it has diverged significantly and now belongs to its own subfamily with calpain-6 [11]. This divergence is also evident within exon 6 alone, where the calpain-5 catalytic domain shows relatively low homology to other human calpains (Figure 4C). Each of the ADNIV mutant residues is conserved in four or fewer of 12 calpain paralogs, suggesting that the residues mutated in ADNIV are specifically important to calpain-5 function and may physiologically distinguish it from the other calpains. The PolyPhen2 sequence analysis program predicted both ADNIV mutations to have damaging effects on protein function (0.999 for Arg243Leu and 0.998 for Leu244Pro) comparable to an active site Cys81Ser mutation (1.0). The SIFT program predicted the Leu244Pro mutation to be comparably pathogenic (0.04) to Cys81Ser (0.03) but predicted the Arg243Leu mutation to be better tolerated (0.1).

To better examine the relationship of ADNIV mutations within the calpain-5 catalytic domain, homology modeling to calpain-2 (m-calpain) was used to generate a three-dimensional structure for calpain-5 (Figure 4D) [12], [13]. Both mutations were outside the active site cleft and relatively far removed from the calcium-binding domains and the binding site of the endogenous inhibitor calpastatin. Interestingly, both the ADNIV-1/2, and ADNIV-3 mutations fell into a region of low electron density, suggesting the presence of a flexible loop (Figure 4E). In calpain-1 (μ-calpain) models, the homologous loop undergoes calcium-induced conformational changes that regulate the proximity of catalytic residues within the active site cleft [14]. This putative loop contains both ADNIV mutants and is highly conserved among all calpain-5 orthologs (Figure 4B).

### Calpain-5 Expression in the Retina

**Figure 5:**
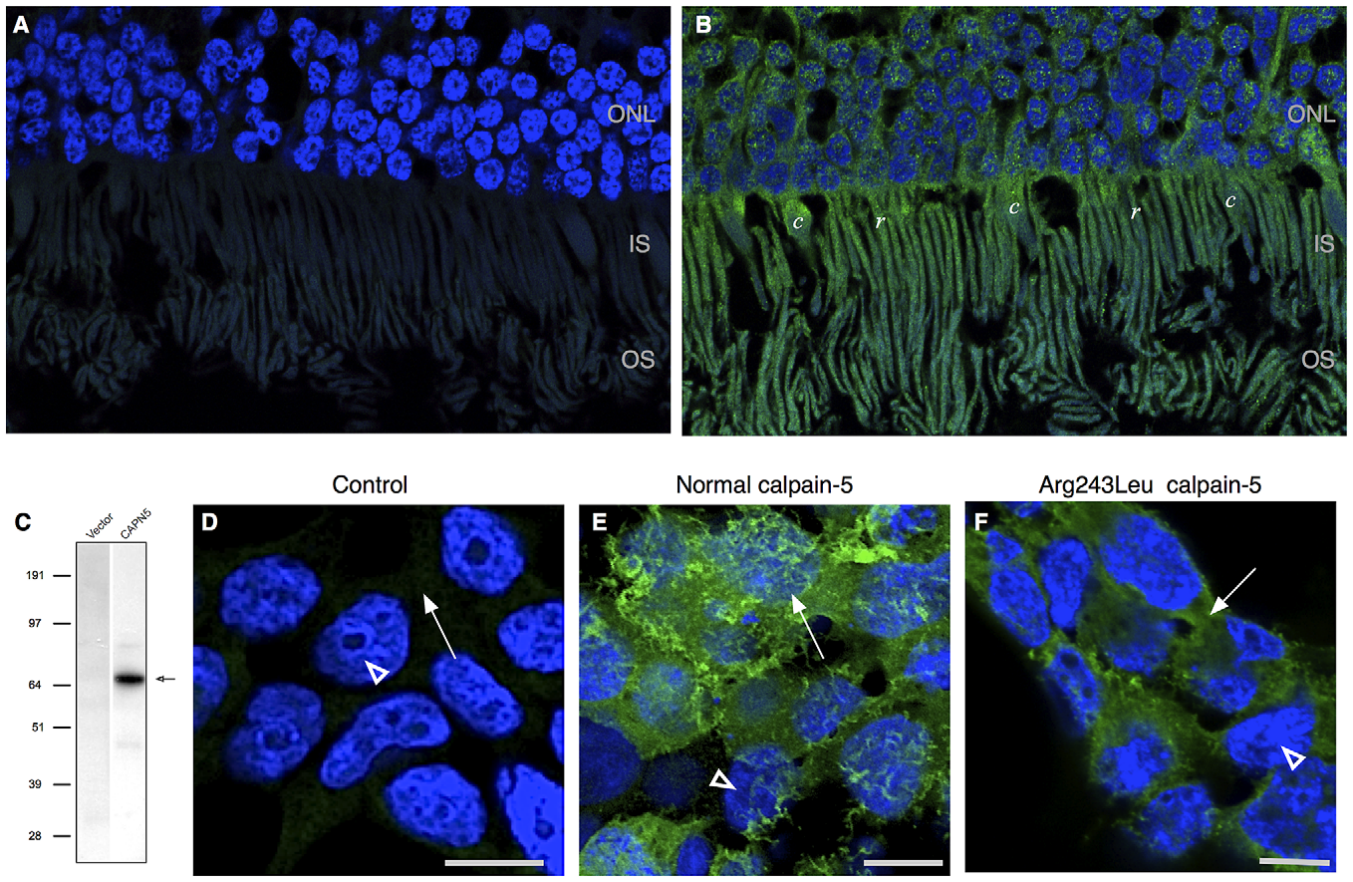
Calpain-5 expression in human retinal photoreceptor cells and cultured cells. A. No significant signal was detected in control retinal sections probed with secondary antibody or primary antibody blocked with recombinant calpain-5. DAPI highlights the cell nuclei (blue) B. Calpain-5 was detected in photoreceptor cells (green). A punctate expression pattern was most prominent overlying the photoreceptor nuclei in the outer nuclear layer (ONL) and inner and outer segments (IS, OS). C. Anti-myc antibody western blot detects a single species (black arrow) of the appropriate size in HEK293T cells transfected with a vector bearing normal, myc-tagged *CAPN5*. D. No significant anti-myc signal was detected in the nuclei (white arrowhead) or cytoplasm (white arrow) of control cells that were treated with transfection reagent alone, vector alone, or secondary antibody alone. E. Anti-myc antibody shows that calpain-5 (green) is expressed in a ruffled pattern (white arrow) that obscures the underlying nuclei (white arrowhead) suggesting a location near the cell surface in these very thin cultured cells. F. Transfection with a mutant *CAPN5* (Arg243Leu) exhibits a more uniform anti-myc signal that does not obscure the nuclei (white arrowhead) and is thus more compatible with localization to the cytoplasm (white arrow).

We evaluated the *CAPN5* transcript in human retinal tissue using RNA sequencing. The transcript was observed at a level of 4.63 fragments per kilobase of exon per million, which places it between the first quartile and the median level of expression for all transcripts observed in the retina. No significant splice variants were detected. Two antibodies against calpain-5 were used to determine whether calpain-5 protein could also be detected in human retinal tissue sections. Both antibodies showed strong calpain-5 expression in the photoreceptor cells (Figure 5A, 5B). There was a punctate pattern of labeling over the nuclei and inner segments with less expression along the outer segments and outer plexiform layer. There was no significant expression in the nerve fiber layer, ganglion cell layer, inner nuclear layer, inner plexiform layer, or retinal pigment epithelium. The localization to the photoreceptor cells is consistent with both the early electrophysiologic abnormalities and the later photoreceptor degeneration seen in ADNIV patients.

### Effect of Calpain-5 Mutations on Intracellular Location

Intracellular compartmentalization is a key regulatory mechanism for calpains [15], [16], [17]. For example, mutations that disturb localized protein interactions with calpain-3 cause limb-girdle muscular dystrophy type 2A [17], [18]. To determine the effect of the ADNIV-causing mutations on the intracellular compartmentalization of calpain-5, HEK293T cells were transfected with normal and mutant *CAPN5* constructs. A western blot with anti-myc antibody revealed a single protein species of the expected size for myc-tagged calpain-5 (Figure 5C). Immunocytochemistry of HEK293T cells showed normal calpain-5 to be localized near the cell surface (Figure 5E). In contrast, both ADNIV mutants were found largely within the cytoplasm (Figure 5F and Figure S2). This suggests that the ADNIV-causing mutations may alter a membrane binding property of the protein.

## Discussion

The calpains are an evolutionarily ancient family of calcium dependent intracellular proteases that utilize a cysteine residue in the active site to mediate limited proteolysis. The multifunctional calpains require careful regulation, since they target multiple intracellular proteins and pathways [15], [16], [17], [19], [20]. Their activity is regulated by intracellular calcium, lipid and protein interactions, subcellular localization, autocatalysis and inhibition by the endogenous peptide calpastatin [18], [20], [21]. There is no consensus amino acid sequence or structural motif that is targeted for cleavage by calpains, and as a result it is often difficult to identify the physiologic substrates of these enzymes, including calpain-5 [22], [23]. *Capn5* is expressed during nematode and mouse embryogenesis [24], [25]. In adults, *CAPN5* is highly expressed in the colon, kidney, liver, trachea, uterus, eye and brain [11], [25]. Calpains have been implicated in the pathogenesis of a wide range of human diseases including cancer, multiple sclerosis, Alzheimer's disease, cataract, diabetes and muscular dystrophy [17], [26]. Some polymorphisms in *CAPN10* and *CAPN5* have been shown to be risk factors for type II diabetes [27], [28]. However, prior to this report, limb girdle muscular dystrophy (LGMD) type 2A was the only disease shown to be caused in a monogenic fashion by variations in a calpain's protein sequence [29].

The evidence that the two missense mutations we observed in *CAPN5* are responsible for ADNIV is compelling. The gene lies within the critical region previously linked to the disease, and all living subjects in the study who are clinically affected were found to harbor a *CAPN5* mutation in exon 6. Each of these two mutations alters an amino acid in the catalytic domain that has been highly conserved throughout evolution. Neither of these mutations was found among any of the clinically unaffected members of the three kindreds we studied, or among thousands of normal individuals.

There are interesting similarities and differences between calpain-associated LGMD and ADNIV. In both disorders, the affected cells (skeletal muscle fibers and photoreceptor cells) experience large changes in membrane potential and intracellular calcium concentration as part of their normal behavior. In both disorders, the non-mutant calpain molecules display functionally critical subcellular localization [17], [18], [20], [30]. A subset of LGMD is associated with leukocyte infiltration into the tissue [31], and all cases of ADNIV are marked by severe intraocular inflammation. The differences in these diseases are also noteworthy. Calpain-associated LGMD is inherited in a recessive fashion and appears to result from loss of calpain-3 function [29]. In contrast, ADNIV is inherited in an autosomal dominant fashion and is caused by missense mutations near the active site. Although these mutations could cause disease through haploinsufficiency, it seems more likely that they result in a gain of function of calpain-5 that causes harm to the photoreceptor cells. *Capn5* knockout mice have no observable phenotype [32], and several human neurological disorders have been associated with excess calpain activity [17], [33], including photoreceptor degeneration [34].

A gain of function mechanism for ADNIV is also supported by the unusual inflammation and neovascularization associated with the disease. There are dozens of monogenic disorders that cause the apoptotic death of photoreceptor cells without causing severe intraocular inflammation or neovascularization of the retina. The latter is much more typical of proliferative diabetic retinopathy than it is heritable photoreceptor disease [8]. It is easier to imagine an unregulated or mislocalized calpain promiscuously activating different signaling pathways, or being released into the extracellular space after photoreceptor death and causing inappropriate angiogenesis and leukocyte recruitment, than it is to imagine a 50% reduction of such a protein causing these dramatic complications.

Whether caused by a gain or loss of function of *CAPN5*, it is likely that the further elucidation of the pathogenic mechanism of ADNIV will provide important new insight into some of the most important causes of irreversible human blindness: autoimmune uveitis, retinitis pigmentosa, proliferative vitreoretinopathy and diabetic retinopathy. The latter condition alone is responsible for as much as 17% of blindness in some regions of the world [4]. The fact that an amino acid change in a single protein can lead to such a phenotype raises the possibility that a common, therapeutically accessible pathway may be shared among these conditions that could be targeted with drugs, antibodies or gene transfer approaches. It is possible that variations in the structure or expression of *CAPN5* cause or modify some of these common disorders and this hypothesis will be important to test in future studies. However, given the extreme phenotypic heterogeneity of these disorders, it will be important to study a large number of subjects in such an experiment, to subdivide the patient cohorts into clinically well-characterized groups, and to screen an equal number of ethnically matched controls for each of these groups.

## Materials and Methods

The study was approved by the University of Iowa's Institutional Review Board and adheres to the tenets set forth in the Declaration of Helsinki. Informed consent was obtained from all study participants. Informed consent was obtained from all study participants.

### Genetic Analysis

Phenotypic ascertainment of the pedigrees included complete ocular examination as previously described [1]. Following genotyping of all adult members of the three ADNIV pedigrees with short tandem repeat polymorphisms within the original disease interval, three members of ADNIV-1 and three members of ADNIV-3 were also genotyped with an Affymetrix GeneChip Human Mapping 50K Array. Exome sequencing was performed using NimbleGen's SeqCap EZ Human Exome v2.0 capture and paired-end (2×100) sequencing on an Illumina HiSeq 2000 instrument at Otogenetics (Norcross, GA). The putative disease-causing mutations in *CAPN5* were evaluated in the ADNIV kindreds and controls using Sanger sequencing and single-strand conformational polymorphism analysis (SSCP) [35]. Sequence analysis of human retinal cDNA was performed using the Illumina HiSeq 2000 instrument.

### Structural Analysis

Primary and secondary structure protein alignments and trees were created with Geneious Pro 5.4.6 (http://www.geneiouspro.com). Yasara Structure (version 11.3.2) was used to generate a homology model of human calpain-5 using calpain-2 (m-calpain) structures (pdb id: 3BOW, 1DF0, 1U5I, 1KFU) as templates [12], [13]. Sequence alignment with the templates was first used to build a backbone model for aligned residues followed by loop modeling and side chain optimization using a combination of steepest descent and simulated annealing minimization. The top ranking of the 20 models generated was used as the homology model of calpain-5. The above steps were automated using Yasara's hm_build macro (http://www.yasara.org). Another homology model was generated using the Phyre server (version 0.2) (http://www.sbg.bio.ic.ac.uk/~phyre), which showed good agreement with the Yasara model for the domain folds and the active site region. The putative alpha-helix region in the Yasara model also formed a helix in the best Phyre model although it was positioned further away from the calpastatin binding site region than the Yasara model. PolyPhen2 (http://genetics.bwh.harvard.edu/pph2/) and SIFT (http://sift.jcvi.org/) sequence analysis software were used to predict the functional effect of mutations.

### RNA Sequencing

RNA sequence analysis was performed by extracting RNA from the retina of a human eye donor using an RNeasy kit from Qiagen (Valencia, CA) according to the manufacturer's instructions, preparing the sequencing library using the Illumina (San Diego, CA) RNA TruSeq sample preparation kit, and sequencing the latter on the Illumina HiSeq 2000 instrument at the Hudson Alpha Institute in Huntsville Alabama. The resulting sequence data were mapped using TopHat [36] and analyzed using Cufflinks [37]. The retinal expression of *CAPN5* was compared to the expression of all other genes expressed in the retina.

### Immunohistochemistry

Donor eyes were received from Iowa Lions Eye Bank (Iowa City, IA). Tissue was fixed in 4% paraformaldehyde solution diluted in 10 mM phosphate buffered saline (PBS), pH 7.4, and 7 µm sections underwent immunohistochemistry (IHC) using a polyclonal anti-calpain 5 primary antibody (Santa Cruz Biotechnology, Inc., Santa Cruz, CA), AlexaFluor 488 donkey anti-rabbit secondary antibody (Invitrogen, Carlsbad, CA) and 4′,6-diamidino-2-phenylindole (DAPI; Invitrogen). Images were captured using an Olympus BX41 microscope equipped with fluorescent filters and the SPOT Advanced software package.

HEK293T cells (ATCC, Manassas, VA) were transfected with normal and mutant *CAPN5* pCMV6-Entry vector plasmids using Turbofectin 8.0 (Origene) transfection reagent according to the manufacturer's instructions. Cells were incubated for 48 hours post transfection. For immunocytochemistry, cells were blocked using 5% bovine serum albumin (Amresco, Solon, OH) diluted in PBS with 0.1% Triton X-100. The polyclonal primary antibody, anti-Myc-tag, was diluted in PBS at 1∶500 and applied to the cells. Alexa Fluor 488 donkey anti-rabbit secondary, at concentration 10 µg/mL, and 0.0001 µg/mL of the counterstain, 4′, 6-diamidino-2-phenylindole (DAPI) (both from Molecular Probes, Eugene, OR), were applied to the cells, Images were captured using a Zeiss LSM 710 equipped with Zen2009 software (Zeiss, New York, NY).

## Supporting Information

Figure S1 Disease stages of ADNIV phenocopy common vitreoretinal conditions. A. In Stage I disease, there are mild cells (white dots) in the vitreous (orange) and a reduced b-wave on electroretinography (ERG). B. In Stage II disease, the anterior chamber (blue) shows mild inflammatory cells and there is early development of cataract. The posterior segment shows moderate cells, retinal pigmentary changes, and some edema in the macula or optic nerve head. There is selective loss of the b-wave in the scotopic bright flash ERG. C. In Stage III disease, the anterior segment shows moderate cells, progressive cataract, and iris synechiae. Progressive inflammation in the posterior segment shows development of vitreous bands and epiretinal membranes, and more posterior retinal pigmentary changes. There is reduction of the a-wave on ERG. D. In Stage IV disease, inflammation in the anterior segment causes neovascular and angle closure glaucoma. Neovascularization develops in the retina with vitreous hemorrhage and progressive retinal detachment that may include features of anterior or posterior proliferative vitreoretinopathy. The ERG becomes non-recordable. E, In Stage V disease, the eye becomes phthisical. (Blue bar, anterior chamber features; orange bar: posterior chamber features; black bar, ERG features) Illustrations by Alton Szeto, MFA.
(TIF)

Figure S2 Calpain-5 mutant expression in cultured cells. Transfection with the ADNIV-3 mutant *CAPN5* (Leu244Pro) exhibits a more uniform anti-myc signal that does not obscure the nuclei (arrowhead) and is thus more compatible with localization to the cytoplasm (arrow).
(TIF)
