# Supplementary figures and images for: Calpain-5 Mutations Cause Autoimmune Uveitis, Retinal Neovascularization, and Photoreceptor Degeneration
Source: PLoS Genet. 2012 Oct 4;8(10):e1003001. doi: 10.1371/journal.pgen.1003001 (PMC3464205; doi:10.1371/journal.pgen.1003001)

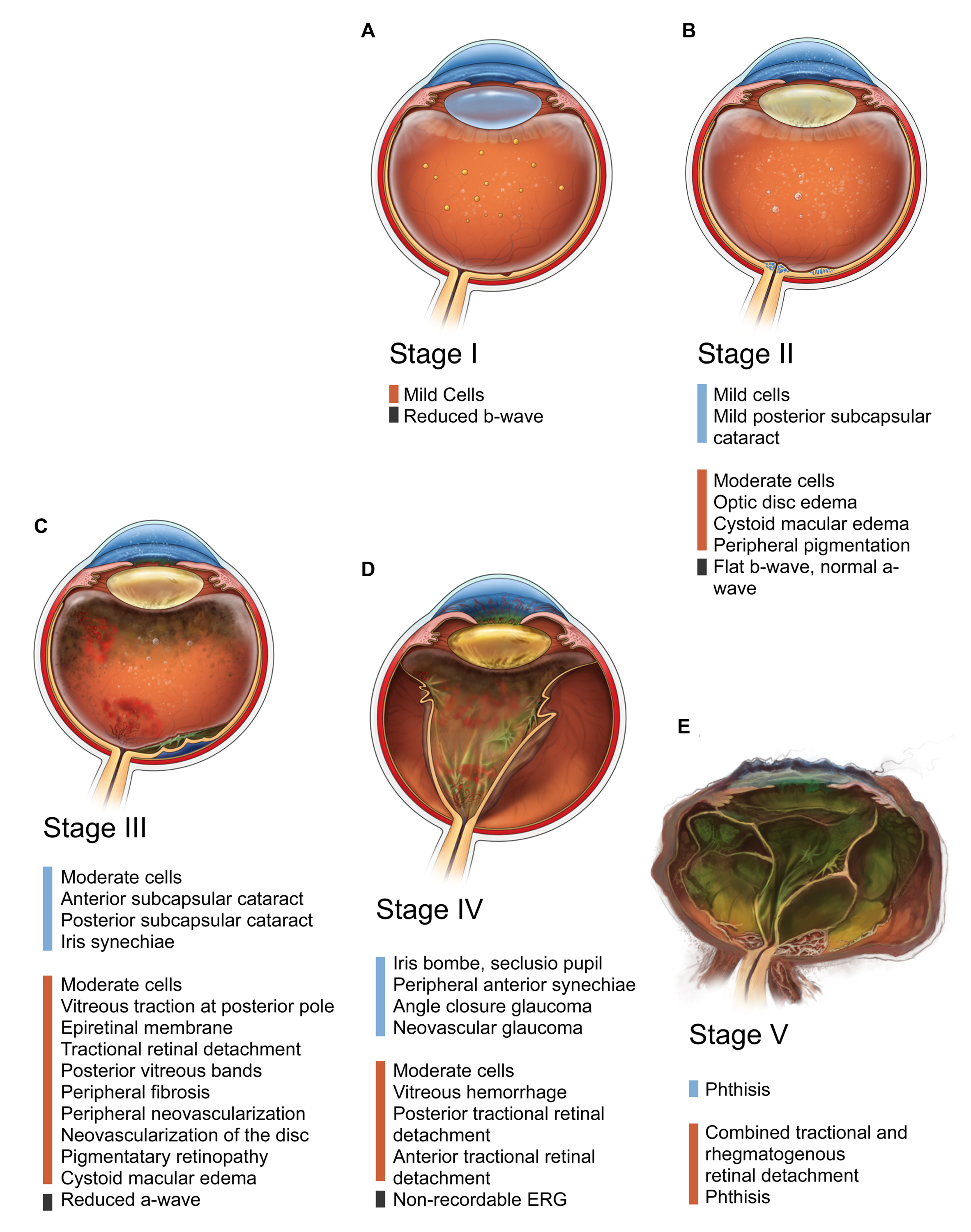

Supplement: Figure S1 — Disease stages of ADNIV phenocopy common vitreoretinal conditions. A. In Stage I disease, there are mild cells (white dots) in the vitreous (orange) and a reduced b-wave on electroretinography (ERG). B. In Stage II disease, the anterior chamber (blue) shows mild inflammatory cells and there is early development of cataract. The posterior segment shows moderate cells, retinal pigmentary changes, and some edema in the macula or optic nerve head. There is selective loss of the b-wave in the scotopic bright flash ERG. C. In Stage III disease, the anterior segment shows moderate cells, progressive cataract, and iris synechiae. Progressive inflammation in the posterior segment shows development of vitreous bands and epiretinal membranes, and more posterior retinal pigmentary changes. There is reduction of the a-wave on ERG. D. In Stage IV disease, inflammation in the anterior segment causes neovascular and angle closure glaucoma. Neovascularization develops in the retina with vitreous hemorrhage and progressive retinal detachment that may include features of anterior or posterior proliferative vitreoretinopathy. The ERG becomes non-recordable. E, In Stage V disease, the eye becomes phthisical. (Blue bar, anterior chamber features; orange bar: posterior chamber features; black bar, ERG features) Illustrations by Alton Szeto, MFA. (TIF) [file pgen.1003001.s001.tif]

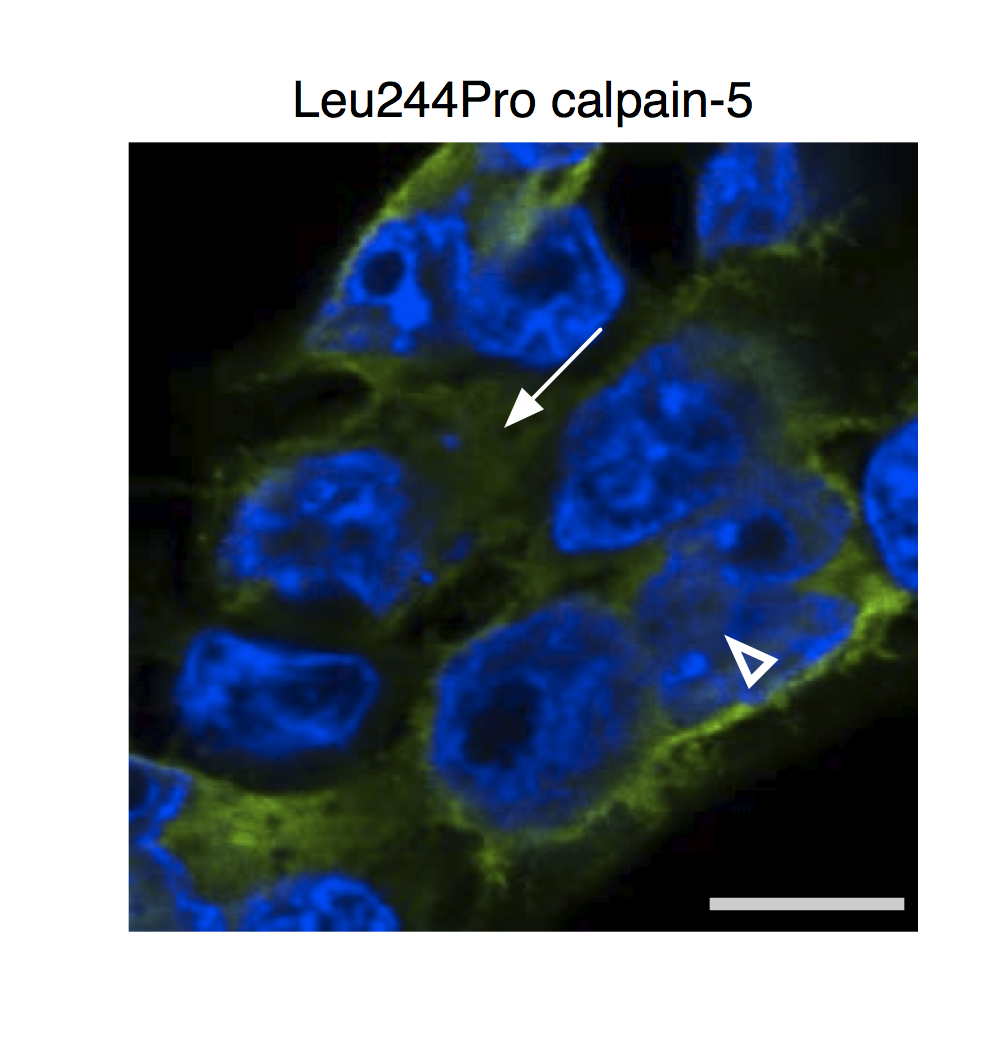

Supplement: Figure S2 — Calpain-5 mutant expression in cultured cells. Transfection with the ADNIV-3 mutant CAPN5 (Leu244Pro) exhibits a more uniform anti-myc signal that does not obscure the nuclei (arrowhead) and is thus more compatible with localization to the cytoplasm (arrow). (TIF) [file pgen.1003001.s002.tif]
